# Highly Efficient Solar Steam Generation by W_18_O_49_@PVA Gels

**DOI:** 10.3390/gels11100783

**Published:** 2025-09-30

**Authors:** Jiefeng Yan, Zhenxing Fang, Jinxing Hu, Yangming Sun, Xinyi Huang, Guannan Zhou, Lu Li, Rui Wang, Yan Chen

**Affiliations:** 1 Ningbo Key Laboratory of Agricultural Germplasm Resources Mining and Environmental Regulation, College of Science and Technology, Ningbo University, Ningbo 315300, China; yanjiefeng@nbu.edu.cn (J.Y.); fangzhenxing128@163.com (Z.F.); hujinxing@nbu.edu.cn (J.H.); 15868324296@163.com (Y.S.); 18968498938@163.com (X.H.); 2 Division of Energy Materials (DNL2200), Dalian Institute of Chemical Physics, Chinese Academy of Sciences, Dalian 116023, China; gnzhouml@163.com (G.Z.); wangrui87@dicp.ac.cn (R.W.); 3 State Key Laboratory of Inorganic Synthesis and Preparative Chemistry, Jilin University, Changchun 130012, China; yanchen@jlu.edu.cn

**Keywords:** oxygen-deficiency, tungsten oxide, NIR absorption, solar steam generation, cross-linking gel

## Abstract

Oxygen-deficient tungsten oxide W_18_O_49_ was synthesized through lattice oxygen escaping at high temperature in N_2_ atmosphere. The temperature and inert atmosphere were critical conditions to initiate the lattice oxygen escaping to obtain W_18_O_49_. The large amount of oxygen vacancies supports its performance in photothermal conversion. The synthesized tungsten oxides were characterized by X-ray diffraction (XRD), scanning electron microscopy (SEM), X-ray photoelectron spectroscopy (XPS), and ultraviolet-visible absorption spectroscopy (UV-Vis). The composite gel was fabricated by the insertion of oxygen-deficient tungsten oxide into PVA-based gel, which was cross-linked by glutaraldehyde. The PVA-based gel ensures a matched water supply speed with that of the evaporation rate due to its hydrophilic nature. The result of the solar steam generation shows that the W_18_O_49_-PVA gel (steam generation rate 2.65 kg m^−2^ h^−1^) was faster than that of the pure PVA gel.

## 1. Introduction

The shortage of freshwater resources and the pressure of energy consumption are increasingly becoming global challenges. Traditional seawater desalination and wastewater treatment technologies often come with high energy consumption and high-cost issues. In recent years, Interfacial Solar Steam Generation (ISSG) technology, characterized by its utilization of green solar energy, low system heat capacity, and high evaporation efficiency, has emerged as a promising sustainable solution [1,2]. The core of this technology lies in the development of efficient photothermal conversion materials and the optimization of evaporator structure design for water/heat management.

ISSG technology achieves efficient capture and conversion of solar energy by localizing photothermal materials at the gas–liquid interface, and maximally confines heat near the evaporation interface, significantly reducing heat loss from the bulk of the water body. The ideal performance relies on three core points: broad-spectrum efficient light absorption, excellent photothermal conversion, and intelligent water/heat management. However, traditional photothermal materials (such as carbon-based materials, noble metal nanoparticles, and semiconductors) may suffer from issues such as narrow light absorption bands, high cost, and insufficient stability [3,4,5]. It is urgent to develop novel, efficient, low-cost, easily integrable material systems that can synergistically optimize light–heat–water transport [6].

The commercial WO_3_ has a band gap of 2.5–2.8 eV which absorbs little visible light [7]. Doping some hetero-valence metal ions or introducing some oxygen vacancies are the common methods to improve the concentration of free-carries so as to enhance light absorption. Tungsten oxide can withstand a large amount of oxygen vacancies so as to allow the formation of various phases. Non-stoichiometric tungsten oxides (WO_3−x_) have attracted increasing interest due to their unique properties [8]. Among these, W_18_O_49_ is a particularly important member of the WO_3−x_ family, which contains the largest amount of oxygen deficiency. Its preferential growth and exceptional chemical stability are extensively considered to make its performance excellent in various fields [9,10,11]. Notably, W_18_O_49_ nanowires are particularly versatile, retaining all the characteristic properties of bulk W_18_O_49_ while also exhibiting a high aspect ratio. This morphology facilitates the efficient transfer of photo-generated carriers along the nanowire axis, which is important for achieving efficient photothermal conversion. Up to now, various methods have been successfully developed to synthesize W_18_O_49_ tungsten oxide, including solvothermal [12], thermal evaporation [13], hydrothermal [14,15], simple reflux [16], microwave-assisted [17], and electron beam irradiation [18] and so on. In this report, the oxygen-deficient tungsten oxide W_18_O_49_ was synthesized through lattice oxygen escaping at high temperature in N_2_ atmosphere.

In order to boost water evaporation efficiency, a fast water supply to the evaporation surface is especially important. Therefore, a hydrophilic network such as PVA should be introduced into the structure of the evaporator. PVA hydrogel could play a crucial dual role as a “water channel” and “thermal barrier” in ISSG evaporators due to its unique three-dimensional network structure [19,20,21,22,23]. Firstly, the PVA network rich in hydroxyl (-OH) groups exhibits strong hydrophilic nature, enabling rapid absorption of water from the underlying layer through capillary forces. Its porous structure provides continuous and low-resistance transmission channels for water molecules, ensuring continuous and sufficient water supply to the evaporation interface and preventing local drying. Secondly, the PVA polymer backbone itself is a poor conductor of heat. The heat generated inside the gel can be used to provide the latent heat required by internal water molecules during evaporation, effectively limiting the diffusion of heat to the bulk of the water body and efficiently “locking” the thermal energy at the evaporation interface, significantly improving heat utilization efficiency [24]. Furthermore, PVA hydrogel possesses excellent flexibility, biocompatibility, and processability (such as freeze–thaw cycling and chemical cross-linking), facilitating the integration of different nanomaterials, and enabling the construction of diversified and adaptable (such as flexible and wearable) evaporator structures. Despite the PPy photothermal conversion material, carbon or another semiconductor with high free carriers (Ti_2_O_3_, CuS_2−x_) is coupled with PVA gel to configure the solar evaporator [20,22,23,25,26,27,28,29]. The morphology of the photothermal conversion material would also impact the solar steam generation performance and the lifetime of the solar evaporator. The preferentially grown W_18_O_49_ with one-dimensional structure would twist around the PVA net structure easily. Therefore, ingenious material design and structural optimization of W_18_O_49_/PVA composite gel can effectively synergize light harvesting, heat generation, water supply, and thermal management, providing a practical approach to achieve ultra-high evaporation efficiency.

## 2. Results and Discussion

**Figure 1:**
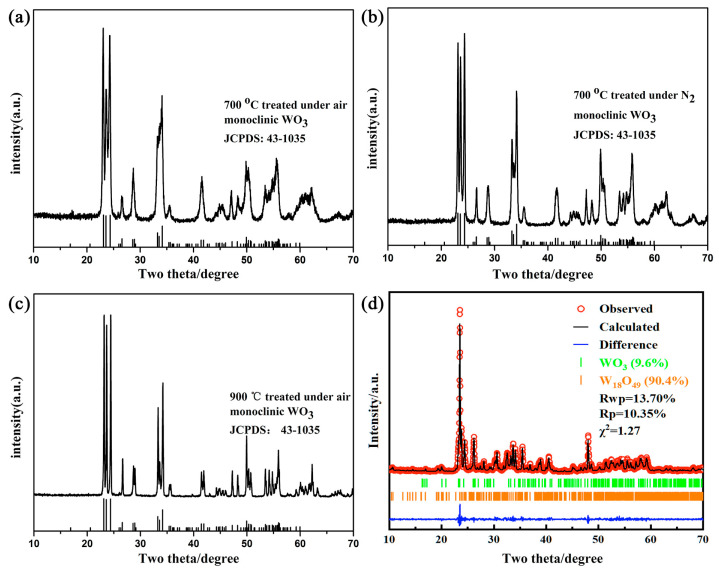
XRD patterns of obtained products with different conditions. (**a**) air 700 °C (**b**) N_2_ 700 °C (**c**) air 900 °C (**d**) N_2_ 900 °C.

Figure 1 shows the XRD patterns of tungsten oxide obtained from various temperatures in air or N_2_ atmosphere. It was important to distinguish that only the XRD pattern of tungsten oxide obtained at 900 °C under N_2_ atmosphere was different from that of three other tungsten oxide samples. At the lower annealing temperature of 700 °C, the XRD patterns of products show monoclinic WO_3_regardless of the air or N_2_ condition, which matches well with the JCPDS No.43-1035. The XRD pattern of tungsten oxide obtained at 900 °C under air condition shows sharper diffraction peaks than that of samples obtained at 700 °C. This is due to the improved crystallinity of the tungsten oxide at higher temperature. Meanwhile, XRD pattern of tungsten oxide obtained at 900 °C under N_2_ condition shows that monoclinic W_18_O_49_ matches well with the JCPDS No.71-2450. This large amount of oxygen vacancies is caused by lattice oxygen escaping and WO_6_ octahedron unit cell rearrangement under inert atmosphere at high temperature. The appearance of the product shows a dark blue which is also widely different from that of other three samples. However, the Rietveld refinement shown in Figure 1d reveals that there were some monoclinic WO_3_ (JCPDS No.43-1035) existing in the obtained W_18_O_49_ (JCPDS No. 71-2450). The residual amount was less than 10%, which demonstrates the successful transformation to the oxygen-deficient tungsten oxide W_18_O_49_.

**Figure 2:**
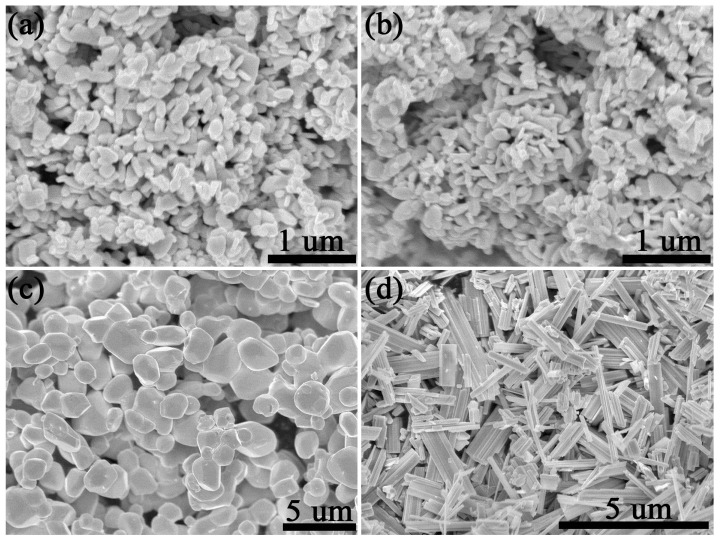
SEM images of tungsten oxide. (**a**) 700 °C air, (**b**) 700 °C N_2_, (**c**) 900 °C air, and (**d**) 900 °C N_2._

Figure 2 shows the SEM images of these tungsten oxides obtained in various conditions. The morphology of tungsten oxide obtained at 700 °C was basically consistent in air or N_2_, which shows nanoplate morphology with a 200 nm radius and 50 nm thickness. When the annealing temperature reached up to 900 °C, the morphology of tungsten oxide turned out to be larger particles with a size larger than 1 um. This is also evidenced by the result of its XRD shown in Figure 1c. The sharper XRD peaks also reveal a larger particle size. It is interesting that the morphology turned out to be nanorods when the annealing treatment was 900 °C in N_2_. This is also consistent with other reported W_18_O_49_ material which have preferential growth in *b* axis direction. It should be noted that N_2_surroundingsaffect the reduced atmosphere of H_2_ or H_2_/N_2_ mixed atmosphere. In a strong reduced atmosphere, a large amount of lattice oxygen in WO_3_ would be eliminated so as to form many other phases such as WO_2.9_, WO_2.83_, or mixed phases and so on [30]. However, in an inert atmosphere, the result shows that the lattice oxygen in WO_3_ is stable even at 700 °C and lattice oxygen escape occurred at 900 °C so as to form W_18_O_49_. Thus, we proposed a new method to synthesize W_18_O_49_ at 900 °C in N_2_.

**Figure 3:**
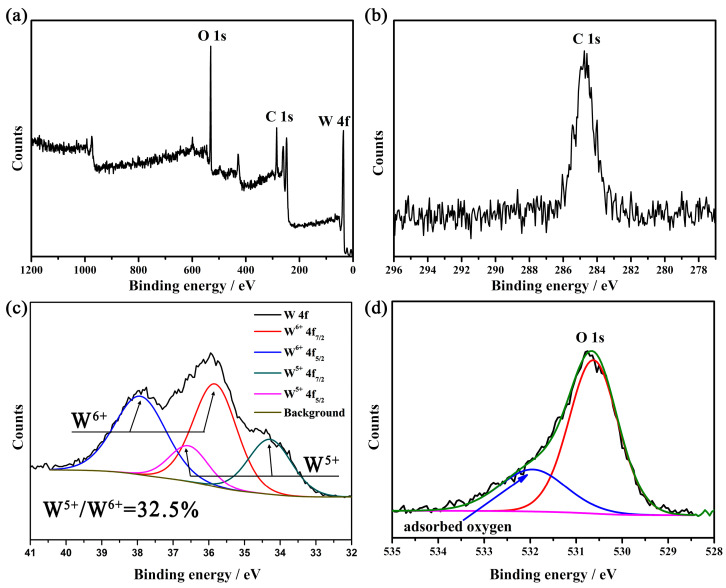
(**a**) XPS survey of tungsten oxide obtained at 900 °C in N_2_, (**b**) C 1s, (**c**) W 4f, (**d**) O 1s.

As the lattice oxygen in tungsten oxide escaped at high temperature in N_2_, large amounts of oxygen defects were generated, which drive the WO_6_ octahedron unit cell rearrangement to form a more stable phase W_18_O_49_. Thus, XPS was conducted to survey the oxidation state of W in the new generated product (shown in Figure 3). Figure 3c shows the XPS spectrum of W 4f orbital in the dark blue sample. The broad shoulder peak in Figure 3 reveals a mixed oxidation state of W. The formation of each oxygen vacancy is assumed to be compensated by two localized electrons on cations (W^5+^) [31]. The doublet peaks at about 37.8 and 35.7 eV are assigned to W 4f_5/2_ and W 4f_7/2_ of W^6+^, respectively. The lower binding energy peaks at 36.4 and 34.3 eV belong to W 4f_5/2_ and W 4f_7/2_ of W^5+^, respectively [32]. The percentage of W^5+^ is calculated to be 32.5% according to the integral areas of the peaks. The XPS spectra of other three tungsten oxide samples do not have a shoulder peak. Its high symmetrical peak reveals the absence of reduced tungsten state (W^5+^). Moreover, Figure 3d shows a large amount of adsorbed oxygen which demonstrates a high concentration of oxygen deficiency. Thus, the temperature and atmosphere are both critical for the synthesis of W_18_O_49_.

**Figure 4:**
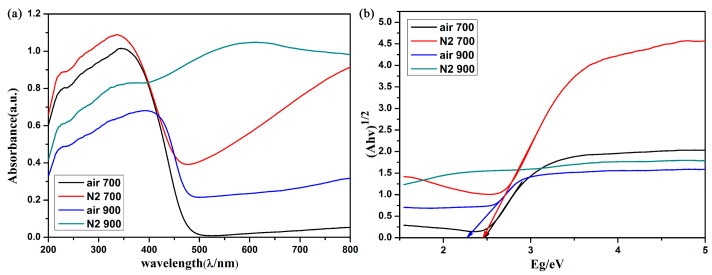
(**a**) Absorption spectra of the tungsten oxide obtained in various conditions, (**b**) calculated band gap of these tungsten oxides.

The appearance of these tungsten oxides obtained in various conditions was extremely varied. The reduced tungsten oxidation state (W^5+^) is often regarded as the color center in tungsten oxide. Therefore, the oxygen-deficient product exhibits a dark blue appearance which differs from that of other tungsten oxides. The optical characterization was converted from diffuse reflectance spectrum by Kubelka–Munk transformation. As shown in Figure 4a, the green line shows a broad absorption during the whole UV-Vis range while the products obtained at 700 °C show only a slight absorption during the visible light range. The red line also exhibits partial absorption in visible range due to its increased crystallinity. The partial red light absorption results in a green appearance of this tungsten oxide powder. The band gap of these tungsten oxides was calculated according to the absorbance. As shown in Figure 4b, the black line and red line represents the sample obtained at 700 °C, demonstrated to be a similar band gap of about 2.5 eV [7,33]. The blue line shows a smaller band gap due to its higher crystallinity. A more orderly arrangement of WO_6_ units causes more absorbance. However, the strong absorbance during the whole UV-Vis range of W_18_O_49_ comes from its high concentration of free carries. These results are similar to the previous reported research; the light absorption capability increases with the increment of free carriers in transition metal oxide semiconductor [34]. A self-floating evaporator with a stable photothermal conversion performance of W_18_O_49_ coupled with carbon foam has been reported and applied in solar steam generation [35]. Here, a fine-designed hydrophilic gel was adopted to play a crucial dual role as a “water channel” and “thermal barrier” so as to significantly increase the evaporation efficiency.

**Figure 5:**
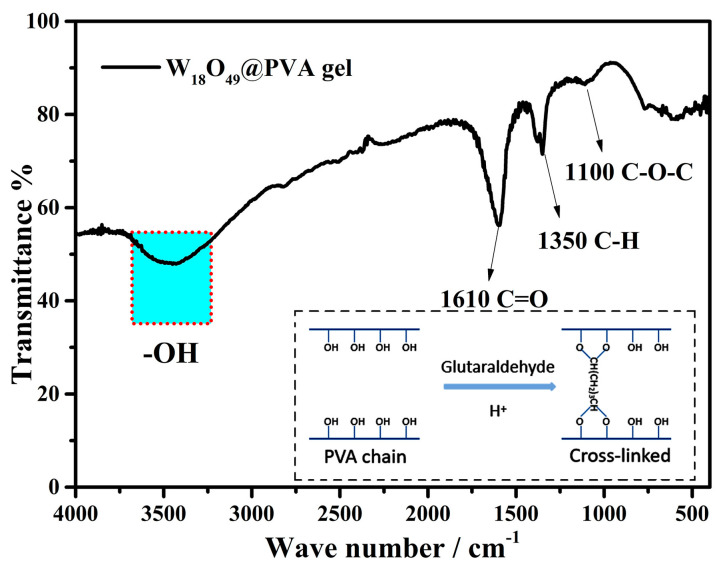
FTIR spectra of the W_18_O_49_@PVA gels.

Figure 5 shows the FTIR spectrum of the composite gel. The PVA chains are rich in hydroxyl groups, which could be cross-linked by glutaraldehyde in acid condition and results in the formation of a three-dimensional interpenetrating network structure [24]. The dialdehyde molecule with a head-to-tail structure is often used to cross-link the long chain polymer with hydroxyl groups [36,37,38]. As PVA dissolves in hot water, the viscosity of the system gradually increases. Therefore, inorganic nanomaterials can be dispersed better in this system to avoid coagulation during the cross-linking process. Thus, this liquid-to-gel process is easy to scale up and costless for preparing the composite gel. This interconnected PVA structure could serve as a thermal barrier in ISSG evaporators due to its low thermal conductivity. Moreover, it also reserves partial hydroxyl groups (as shown the strong absorption at ~3500 cm^−1^). This reservation of hydroxyl groups ensures its hydrophilicity which is critical to the water transportation in ISSG technology. Therefore, the composite gel plays a crucial dual role as a “water channel” and “thermal barrier” in ISSG evaporators due to its unique three-dimensional network structure.

**Figure 6:**
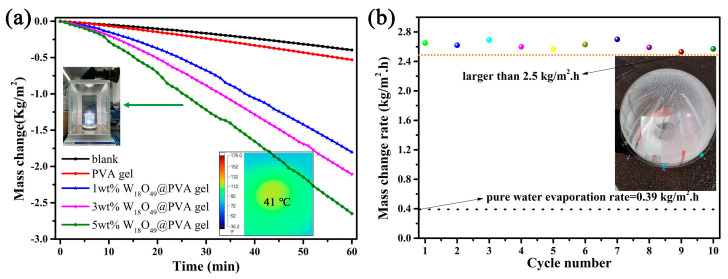
(**a**) Solar steam generation test under 1 sun irradiation (inserted image was the W_18_O_49_@ PVA gel irradiated by simulated solar light and the thermal image recorded by IR camera). (**b**) The recyclability of the 5 wt% W_18_O_49_@ PVA gel by 10 cycles and its outdoor experiment image.

**Figure 7:**
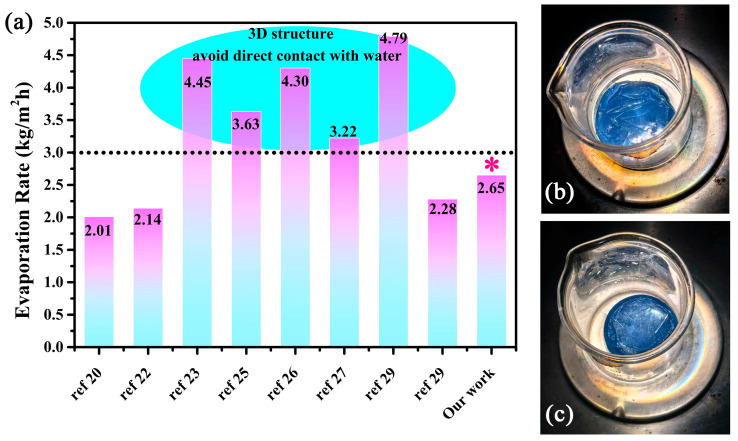
(**a**) A quantitative comparison of the evaporation rate among the reported solar evaporators. (**b**) The image of composite gel in 10 wt% salt solution before evaporation. (**c**) The image of composite gel in 10 wt% salt solution under continuous 6 h irradiation. The asterisk (*) indicates our work.

The solar steam generation test was conducted under 1 sun irradiation to estimate the practical performance of this well-designed composite gel. The mass change represents the amount of evaporated water, which was recorded at 1 min intervals. It is apparent that the vapor generation with composite gel is more efficient than that of pure water or pure PVA gel. The black line represents the evaporation rate of pure water, which was calculated to be 0.39 kg/m^2^. The evaporation rate of the cross-linked PVA gel was 0.53 kg/m^2^ which is faster than that of pure water. The heat loss decreases due to its polymer nature of low thermal conductivity. Meanwhile, the surface heat generation was limited due to the lack of effective photothermal conversion material. When the PVA gel coupled with oxygen-deficient rich material W_18_O_49_, the vapor generation rate was remarkably improved. Furthermore, the evaporation rate also accelerates as the amount of W_18_O_49_ increases. The max evaporation rate of 5 wt% W_18_O_49_@PVA gel reaches up to 2.65 kg/m^2^, which is more than 6 times that of pure water. The temperature of this evaporation surface is less than 41 °C due to the fast water evaporation (see inset picture in Figure 6a). This also reveals that the localized heat generation was used in the phase change in water and there is not much heat loss. Moreover, the long-term stability test was conducted for 10 cycles under the same irradiation condition. The result in Figure 6b shows a good recycling performance, in which the lowest evaporation rate was larger than 2.5 kg/m^2^·h. It was also easy to scale up due to the uncomplicated processing of the cross-linking between PVA and dialdehyde. A 12 cm diameter composite gel was fabricated to demonstrate its outdoor performance (surrounding temperature near 30 °C). The inset image in Figure 6b shows a large number of droplets on the inner wall of the transparent spherical dome. It could be concluded that a suitable water supply coupled with the localized heat generation results in a high solar utilization and water evaporation rate. This fast evaporation rate benefitted from the reduced vaporization enthalpy, which comes from the water cluster theory. This theory reveals that water can be evaporated as either a single molecule or in small clusters consisting of a few to tens of molecules. According to the calculation of the reported literature (also see in experimental details), the water vaporization enthalpy of the W_18_O_49_@PVA gel decreases from 2400 to 2020 kJ/kg [25]. The solar evaporation efficiency was calculated to be 93.6%. Thus, this hydrophilic PVA gel plays two key roles as a “water channel” and “thermal barrier”, which results in a reduced vaporization enthalpy so as to improve the water evaporation rate. There were some reported solar evaporators with a3D structure with indirect contact with water exhibiting excellent water evaporation rates which were all larger than 3.0 kg/m^2^·h. However, the indirect contact with water would inevitably cause salt precipitation which could cause a dramatic decrease in the long-lasting duration. Direct contact with water would solve this problem. The precipitated salt would dissolve when contacted with bulk water. As shown in Figure 7a,b, the surface of the composite gel had no obvious salt deposition after 6 h continuous irradiation in high salt concentration solution (10 wt%). The fine salt resistance performance also demonstrates a fast water supply capability of this composite gel. Among the gels which had direct contact with water, the W_18_O_49_@PVA gel has shown a relatively high evaporation efficiency.

## 3. Conclusions

A highly efficient interfacial solar steam generation system was well-designed by inserting W_18_O_49_ into PVA base gel. The photothermal conversion material of W_18_O_49_ was successfully synthesized through direct annealing at 900 °C in N_2_. The PVA gel still keeps its hydrophilic nature after cross-linking with glutaraldehyde, which is essential for its water transportation. The composite W_18_O_49_@PVA gel exhibits a fast water evaporation rate 2.65 kg/m^2^ which is more than 6 times that of pure water. This enhancement is attributed to the coupling-efficient light absorption and heat conversion properties of W_18_O_49_ nanorods and the water transportation of PVA gel, which significantly improve the overall performance of the composite gel. This composite gel should have promising applications in the water purification field.

## 4. Materials and Methods

### 4.1. Materials

Tungsten (VI) oxide, WO_3_ (purity lager than 99.8%) was purchased from Aladdin (Shanghai, China). Poly(vinyl alcohol) (PVA) M_w_ = 31,000–50,000 and glutaraldehyde 50% in water (Analytical Reagent) were also purchased from Aladdin (Shanghai, China). There was no need to purify before use. HCl was diluted to 1M to catalysis the cross-linking reaction. High purity N_2_ (99.999%) was purchased from CixiQunying Gas Co., Ltd. (Cixi, China). Deionized water (conductivity less than 10 μS/cm) was self-made to wash the gel.

### 4.2. Procedures of the Composite Gel Synthesis

#### 4.2.1. The Synthesis of Tungsten Oxide

Position the commercial yellow tungsten trioxide powder at the center of the tubular furnace. Initially, purge high-purity (99.999%) nitrogen into the tube for 30 min to extrude the air. Subsequently, heat the powder to the setting temperature at a ramping rate of 10 °C/min and keep for 2 h. After allowing it to cool down to room temperature, oxygen-deficient tungsten oxide product was obtained. For the purpose of comparing the impact of different atmospheres on the products, tungsten oxide materials were also prepared under identical heat treatment conditions in air for comparative analysis. The prepared series products were labeled as N_2_-700, N_2_-900, Air-700, and Air-900 for different tungsten oxide, respectively. The Air-700 and Air-900 tungsten oxide are yellow powders and the N_2_-700 and N_2_-900 show a pine green and dark blue appearance, respectively.

#### 4.2.2. Preparation of PVA Gel

PVA (1 g), glutaraldehyde (125 μL, 50%wt in DI water), and Dl water (10 mL) were mixed together by sonication (solution C). Then, HCl (50 μL, 1.0 M) was added to 10 mL of solution C, the gelation was carried out for 12 h, and the obtained cross-linked gel was immersed into DI water overnight to remove the impurities. The purified composite gel was frozen by liquid nitrogen and then thawed in DI water at a temperature of 30 °C. The freezing–thawing process was repeated for 5 times. Finally, the obtained HNG sample was freeze-dried.

#### 4.2.3. Preparation of W_18_O_49_@PVA Composite Gel

In a typical synthesis, PVA (1 g), glutaraldehyde (125 μL, 50%wt in DI water), and Dl water (10 mL) were mixed together by sonication (solution C). Then, HCl (50 μL, 1.0 M) and a certain amount of W_18_O_49_ (1 wt%, 3 wt% and 5 wt% according to the mass of PVA) were added to 10 mL of solution C, the gelation was carried out for 12 h, and the obtained composite gel was immersed into DI water overnight to remove the impurities. The purified composite gel was frozen by liquid nitrogen and then thawed in DI water at a temperature of 30 °C. The freezing–thawing process was repeated for 5 times. Finally, the obtained HNG sample was freeze-dried.

#### 4.2.4. Experiment of Solar Steam Generation

The solar steam generation test was conducted at 25 °C and irradiated by simulated solar light (AM1.5 without condensing lens, irradiation power 1 kW/m^2^). The distance from the gel surface to the simulated light was kept at 20 cm. The diameter of the composite gel was 4 cm and the thickness was 2 cm. The water level in the beaker was kept almost the same with the surface of gel (no more than the gel’s surface to avoid large heat loss). The outer space of the container was completely in contact with the surroundings with no air flow (30 °C, humidity 50–60%), without the covering of thermal insulation foam. The mass change was recorded at 1 min intervals during the solar irradiation. The mass change in the system without irradiation was also recorded at 1 min intervals to represent the dark-condition water evaporation. A thermal camera was used to record the gel surface temperature, which directly demonstrates the water evaporation effect.

The reduced water evaporation enthalpy was calculated by the first law of thermodynamics (*ΔH_w_m_w_* = *ΔH_g_m_g_*). *ΔH_w_* and *ΔH_g_* are the evaporation enthalpies of pure water and wet composite hydrogel, respectively. The *m_w_* and *m_g_* refer to the weight change rates of pure water and wet composite hydrogel. The weight change in composite hydrogel and pure water with the same surface area was recorded without irradiation (surrounding temperature was 30 °C) during 60 min. The mass change of pure water and the wet composite gel was 0.0200 and 0.0240 g, respectively.

### 4.3. Characterization

The XRD patterns were recorded by using PANalytical B.V. Empyrean X-ray powder diffraction (Malvern Panalytical, Enigma Business Park, Grovewood Road, Malvern, WR14 1XZ, UK) with Cu Kα radiation over a range of 10–70° (2θ) with 0.02° per step, scanning rate 10°/min. SEM images were obtained with a JSM-6700F electron microscope (JEOL, 1-2 Musashino 3-chome, Showa City, Tokyo, Japan), operating voltage 5 kV, working current 10 mA. X-ray photoelectron spectrometer was recorded by ESCALAB 250 (Thermal Fisher, Waltham, MA, USA). UV-Vis spectra was recorded by Lambda 950 (PerkinElmer, Waltham, MA, USA). The FTIR spectrum was recorded by Fourier Transform Infrared Spectrometry FTIR-650 (Beijing, China). The mass change was recorded by electronic analytical balance (FA2004, Shanghai, China).

## Data Availability

The data presented in this study are openly available in article.
